# Disclosing the Antioxidant and Neuroprotective Activity of an Anthocyanin-Rich Extract from Sweet Cherry (*Prunus avium* L.) Using In Vitro and In Vivo Models

**DOI:** 10.3390/antiox11020211

**Published:** 2022-01-22

**Authors:** Monica Filaferro, Alessandro Codeluppi, Virginia Brighenti, Francesca Cimurri, Ana María González-Paramás, Celestino Santos-Buelga, Davide Bertelli, Federica Pellati, Giovanni Vitale

**Affiliations:** 1 Department of Life Sciences, University of Modena and Reggio Emilia, Via G. Campi 103/287, 41125 Modena, Italy; monica.filaferro@unimore.it (M.F.); alessandro.codeluppi@unimore.it (A.C.); virginia.brighenti@unimore.it (V.B.); 205168@studenti.unimore.it (F.C.); davide.bertelli@unimore.it (D.B.); 2 Grupo de Investigación en Polifenoles, Unidad de Nutrición y Bromatología, Facultad de Farmacia, Universidad de Salamanca, Campus Miguel de Unamuno, 37007 Salamanca, Spain; paramas@usal.es (A.M.G.-P.); csb@usal.es (C.S.-B.)

**Keywords:** *Prunus avium* L., sweet cherry, polyphenols, anthocyanins, oxidative stress, neurodegeneration, *Drosophila melanogaster*, *Caenorhabditis elegans*

## Abstract

In this study, an autochthonous variety of sweet cherry (*Prunus avium* L.), namely “Moretta di Vignola”, was processed to prepare extracts rich in polyphenols, which were characterized by high-performance liquid chromatography (HPLC) separation coupled to UV/DAD and ESI-MS^n^ analysis. Then, a sweet cherry anthocyanin-rich extract (ACE) was prepared, fully characterized and tested for its activity against Parkinson’s disease (PD) in cellular (BV2 microglia and SH-SY5Y neuroblastoma) and in *Drosophila melanogaster* rotenone (ROT)-induced model. The extract was also evaluated for its antioxidant activity on *Caenorhabditis elegans* by assessing nematode resistance to thermal stress. In both cell lines, ACE reduced ROT-induced cell death and it decreased, alone, cellular reactive oxygen species (ROS) content while reinstating control-like ROS values after ROT-induced ROS rise, albeit at different concentrations of both compounds. Moreover, ACE mitigated SH-SY5Y cell cytotoxicity in a non-contact co-culture assay with cell-free supernatants from ROT-treated BV-2 cells. ACE, at 50 µg/mL, ameliorated ROT (250 μM)-provoked spontaneous (24 h duration) and induced (after 3 and 7 days) locomotor activity impairment in *D. melanogaster* and it also increased survival and counteracted the decrease in fly lifespan registered after exposure to the ROT. Moreover, heads from flies treated with ACE showed a non-significant decrease in ROS levels, while those exposed to ROT markedly increased ROS levels if compared to controls. ACE + ROT significantly placed the ROS content to intermediate values between those of controls and ROT alone. Finally, ACE at 25 µg/mL produced a significant increase in the survival rate of nematodes submitted to thermal stress (35 °C, 6–8 h), at the 2nd and 9th day of adulthood. All in all, ACE from Moretta cherries can be an attractive candidate to formulate a nutraceutical product to be used for the prevention of oxidative stress-induced disorders and related neurodegenerative diseases.

## 1. Introduction

Oxidative stress is characterized by an unbalance in reactive oxygen species (ROS) production and antioxidant surveillance system that results in an intracellular accumulation of these radicals [1,2,3]. In this context, oxidative stress is considered to be an important and common mechanism underlying several chronic and degenerative diseases, like diabetes, cardiovascular diseases, chronic intestinal inflammation, cancer, and neurodegenerative disorders. Therefore, the modulation of oxidative stress represents a relevant target for prevention or treatment of these diseases [4,5,6].

With regards to neurodegenerative diseases, like Parkinson’s disease (PD) and Alzheimer’s disease (AD), there is robust scientific evidence indicating that several ROS-mediated pathways may be involved in the pathology development. Indeed, it has been demonstrated that the accumulation of iron in the brain brings to higher ROS generation, involvement of mitochondrial pathways and to a reduction in endogenous antioxidant levels [7]. The brain is particularly exposed to oxidative stress, due to its elevated oxygen consumption and high content of polyunsaturated fatty acids. PD is a progressive neurodegenerative disease that leads to impaired motor function. It is characterized by a loss of dopaminergic neurons in the *substantia nigra* and it is second only to AD in its prevalence [8]. Increasing evidence suggests that elevated oxidative stress and neuroinflammation associated with microgliosis may be responsible for dopaminergic neuronal atrophy and, ultimately, the clinical manifestation of PD [9,10]. Conventional pharmacological therapies for PD patients mainly replenish striatal dopamine, providing symptomatic relief only. Hence, there is an urgent need for novel therapeutic compounds with neuroprotective activity to be employed as independent or adjunctive therapy along with dopamine replacement therapy in PD. In this context, natural antioxidant agents, such as polyphenols, play an important role. Polyphenols can act as direct antioxidants either by preventing or scavenging ROS or indirectly by enhancing the activity of endogenous antioxidant defenses [1,2,3,11]. Anthocyanins are a class of polyphenols responsible for the red to violet color of several fruits and they are considered potent natural antioxidants [12,13]. Due to their peculiar molecular structure, anthocyanins are able to directly scavenge ROS or prevent their generation thanks to their metal chelation properties [14]. These molecules are recognized with remarkable health properties, namely in the prevention of pathologies such as cardiovascular and degenerative disorders [15,16].

*Prunus avium* L. (sweet cherry) is the source of widely consumed fruits, which constitute a rich source of phenolic compounds, in particular anthocyanins [17,18,19,20]. Among all cherries, some cultivars are less appreciated by the consumer due to their small size and they have been in part considered as a food supply chain waste [17,18,19,20,21]. The cultivation of the “Moretta di Vignola” cherry variety has ancient origins and it is well rooted in the Emilia-Romagna region (Italy). Moretta fruits have either a medium or small size and, thanks to their content in sugars, vitamins, and mineral salts, they can be considered as a nourishment with a very high nutritional value, also suitable for children, elderly people, and athletes.

Previous studies revealed that the polyphenolic content of Moretta fruits is far superior to other common cherry cultivars [22]. Considering the results obtained, it is also necessary to underline how the Moretta cherry has phenolic contents absolutely comparable to those of fruits much better acknowledged for their beneficial properties as linked to polyphenols, such as small red fruits (blueberries, blackberries, etc.) [22]. The anthocyanin composition of Moretta cherry is characterized by the presence of cyanidin glycosides, followed by rutinosides of peonidin and delphinidin, thus generating a unique profile “chemical fingerprinting” that distinguishes the Moretta from other cultivars considered [23]. Moreover, these characteristics assume particular importance considering that the different classes of phenolic substances that can be found in the fruit increase their activities by synergistic action. 

The correlation between phenolic profile of sweet cherry (fresh weight, FW) and its antioxidant properties has been also investigated by Acero et al. in different Spanish local cultivars, evidencing that the phenolic composition may significantly differ from one variety to another, resulting in different capacities in radical scavenging [23]. Similar studies have been carried out with different sweet cherry cultivars from other countries [24,25,26,27]. The fruit quality characteristics, phenolic compounds and antioxidant capacities of several sweet cherry cultivars from Sicily (Italy) were also evaluated by Ballistreri et al., who have highlighted the high level of phenolic compounds and antioxidant capacity of some sweet cherry fruits, which implied that they might be sources of bioactive compounds that are relevant to human health [28].

In the light of all the above, the aim of this work was to explore the role of bioactive compounds from Moretta sweet cherries against neurodegeneration (PD models) and oxidative stress. Thus, the phytochemical profile of the fruit extracts was analyzed in terms of phenolic compounds by means of high-performance liquid chromatography (HPLC) coupled with UV/DAD and ESI-MS^n^ detection. An anthocyanin rich extract (ACE) from Moretta cherries was fully characterized and tested: (1) to evaluate its viability modulation and antioxidant effect in murine microglia (BV-2) and human neuroblastoma (SH-SY5Y) cells; (2) to assess its neuroprotective effects against neurotoxin-induced cytotoxicity in cellular PD model; (3) to study its neuroprotective effects in *Drosophila melanogaster* model of chemically induced PD; and (4) to examine its capacity to improve the resistance of *Caenorhabditis elegans* against thermal stress.

## 2. Materials and Methods

### 2.1. Chemicals and Solvents for Fruit Extraction and Analysis

Methanol (MeOH), ethyl acetate (EtOAc), and formic acid (HCOOH), all HPLC grade, hydrochloric acid (HCl) 37%, sodium carbonate (Na_2_CO_3_), aluminum chloride (AlCl_3_), potassium chloride (KCl), sodium acetate, gallic acid (GA), quercetin dihydrate (QE), acetic acid, Folin–Ciocalteu reagent and reversed-phase C_18_ resin (40–63 µm) were purchased from Merck (Darmstadt, Germany). Water (H_2_O) was purified using a Millipore system (Burlington, MA, USA).

### 2.2. Chemicals and Material for Biological Assays

Murine BV2 microglia cells were kindly provided by Elisabetta Blasi, University of Modena and Reggio Emilia, Modena, Italy. Human neuroblastoma SH-SY5Y cells were purchased from the European Collection of Authenticated Cell Cultures (ECACC), Dulbecco’s Modified Eagle Medium: F-12 Nutrient Mixture Hank (DMEM-F12 1:1) and Hank’s Balanced Salt Solution (HBSS) were obtained from Gibco (Thermo Fisher Scientific, Waltham, MA, USA), RPMI 1640 and RPMI 1640, no phenol red, were obtained from Corning (Corning, New York, NY, USA), non-essential amino acids, L-glutamine, Foetal Bovine Serum (FBS), penicillin/streptomycin solution, Rotenone (ROT), DMSO, Thiazolyl blue formazan (MTT), sodium dodecyl sulfate (SDS), agar, cholesterol, sodium ampicillin, nystatin, bacto-yeast, 5-fluoro-2′-deoxyuridine (FUdR), phosphate buffered saline (PBS), peptone enzymatic digest from soybean, bacto-tryptone, sodium chloride, magnesium sulphate calcium chloride, monopotassium phosphate, dipotassium phosphate, sodium hydroxide, di-sodium hydrogen phosphate, sodium hypochlorite, Tween 20 were purchased from Merck (Darmastadt, Germany), H2DCFDA (2,7 dichlorodihydrofluorescein diacetate) was from Invitrogen (Thermo Fisher Scientific, Waltham, MA, USA). White eyed (*w1118*) strain flies were obtained from the Bloomington Stock Center (Department of Biology, Indiana University, Bloomington, IN, USA), Jazz-Mix Drosophila Food was from Fisher (Thermofisher, Waltham, MA, USA), yeast *Kluyveromyces fragilis* was from VWR (VWR Chemicals, Milan, Italy). *C. elegans* wild type N2 strain and *Escherichia coli* OP50 were obtained from the *Caenorhabditis* Genetics Centre at the University of Minnesota (Minneapolis, MN, USA).

### 2.3. Plant Material

Cherry fruits belonging to the autochthonous Italian variety “Moretta di Vignola” were considered. Samples were harvested in spring 2018 in the countryside nearby Modena (Italy). Fruits were stored at −20 °C until their use.

### 2.4. Preparation of Cherry Extracts

About 20 g of frozen cherries was cut into smaller pieces and deprived of the seeds. The remaining pulp was placed into a beaker, allowed to thaw, and extracted with 30 mL of a MeOH-0.5 N HCl (95:5, *v*/*v*) by means of dynamic maceration at room temperature (r.t.) for 1 h. The extract was then centrifuged at 4000× *g* for 10 min and paper filtered. The residue was re-submitted to two additional extraction procedures with aliquots of 30 and 20 mL of solvent for 30 min, centrifuged, and paper filtered. The combined extract was finally brought to the volume of 100 mL and used for the determination of total polyphenols and total anthocyanins. To facilitate the reading, this extract will be mentioned as total cherry extract (TCE) throughout the paper.

Additional 20 g of frozen cherries were extracted as described above, using EtOAc. In this way, a flavonoid rich cherry extract (FCE), deprived of the more polar anthocyanins, was obtained and used for colorimetric determination of total flavonoids.

### 2.5. Preparation of the Anthocyanin-Rich Cherry Extract

A portion of 250 g of frozen cherries was cut into smaller pieces and deprived of the seeds. The remaining pulp was placed into a beaker, allowed to thaw, and extracted with 100 mL of a MeOH-0.5 N HCl (95:5, *v*/*v*) by means of an ultrasound-assisted extraction (UAE) at r.t. for 1 h. The extract was then centrifuged at 4000× *g* for 10 min and paper filtered. The residue was again submitted to the extraction procedure for other five times. The extracts were finally collected and concentrated under vacuum using a rotary evaporator. 

The crude extract was further purified using a pad of reversed-phase C_18_ resin [29]. The resin was suspended in acetone, placed in a Buchner funnel, and conditioned with H_2_O. The extract was slowly laid in small aliquots on the resin layer and then gently washed with approximately 2 L of H_2_O, thus removing sugars and more polar substances. Finally, MeOH-0.5 N HCl (95:5, *v*/*v*) was added to elute anthocyanins from the C_18_ resin. The acidic eluate rich in anthocyanins was concentrated under vacuum and re-dissolved in H_2_O for a freeze-drying procedure. The final anthocyanins-rich extract obtained after lyophilization process will be further mentioned as anthocyanin rich cherry extract (ACE).

### 2.6. Total Polyphenol Content of Cherry Extracts

The extract obtained by the above-mentioned procedures (Section 2.4 and Section 2.5) were checked for their total content of polyphenols by means of the Folin–Ciocalteu’s assay. Total polyphenols (TP) content was expressed as mg of GA equivalents per gram (mg/g) of both the fresh fruit (FW) and ACE. For this purpose, 50 μL of TCE and of a 0.5 mg/mL solution of ACE in acidified MeOH were added with 2.5 mL of Folin–Ciocalteu reagent (diluted 1:10 with H_2_O) and 2.0 mL of a saturated Na_2_CO_3_ solution. The blue-colored solution obtained was kept at room temperature and away from light for 2 h. In parallel, standard solutions of GA were prepared ranging from 1.0 to 0.05 mg/mL. Absorbance values at λ 760 nm of the sample and standard solutions were determined using an UVmini-1240 UV/Vis spectrophotometer (Shimadzu, Milan, Italy). A four-point external calibration curve was generated by plotting the concentration of GA standard solutions versus their absorbance at λ 760 nm. A blank solution was also prepared, containing all the chemicals but the cherry extract. Two spectrophotometric measurements were performed for each solution.

### 2.7. Total Anthocyanin Content of Cherry Extracts

Total anthocyanin (TA) content was determined following the pH differential method, as described in the literature [30] and expressed as mg of cyanidin-3-*O*-glucoside equivalents per gram (mg/g) of both the fresh fruit (FW) and ACE. For what concerns ACE, 10 mg were dissolved in 1 mL of MeOH:HCl 0.5 N (95:5, *v*/*v*). Then, two aliquots of either the acidic methanol or lyophilized cherry extract were properly diluted with either KCl (0.025 M, pH 1) or sodium acetate buffer (0.4 M, pH 4.5) and allowed to equilibrate for 15 min prior spectrophotometric determinations. Absorbance measurements of pH 1 and pH 4.5 extract solutions were carried out at both 510 nm (λ_max_ of cyanidin-3-*O*-glucoside) and 700 nm to correct for haze against a blank cell filled with distilled H_2_O. 

The absorbance of diluted samples (A) was calculated as follows:

A = (A_510_ − A_700_)_pH1_ − (A_510_ − A_700_)_pH4.5_

TA content was calculated according to the following equation:

$$mg/g=\frac{A\times MW\times DF}{\varepsilon \times g}$$

where: *A* is the absorbance calculated as indicated above, *MW* is the molecular weight of cyanidin-3-*O*-glucoside, *DF* is the dilution factor applied to the extract, *ε* is the molar absorptivity of cyanidin-3-*O*-glucoside, and *g* is the weight of the lyophilized extract expressed in grams. For each solution, the absorbance at λ 510 and λ 700 nm was determined twice.

### 2.8. Total Flavonoid Content of Cherry Extracts

Total flavonoid (TF) content was estimated by means of a colorimetric assay [31]. TF content was expressed as total mg of QE equivalents per gram (mg/g) of both the fresh fruit (FW) and ACE. An aliquot of 10 mL of FCE was brought to dryness and re-dissolved in 1 mL of MeOH. A portion of 20 mg of ACE was extracted five times with 1 mL of EtOAc. Extracts were filtered, collected, and brought to dryness. The residue was re-dissolved in 1 mL of MeOH. A volume of 400 µL of both MeOH extracts were added with 200 µL of a 5% solution of AlCl_3_ in H_2_O and brought to the final volume of 10 mL with MeOH. The yellow-colored solution was then allowed to stand for 30 min prior spectrophotometric determination. In parallel, standard solutions of quercetin (QE) were prepared ranging from 8.0 to 0.05 µg/mL. The same sample preparation procedure followed for cherry extracts was applied to quercetin standard solutions. The absorbance at λ425 nm of the sample and standard solutions was determined using an UVmini-1240 UV/Vis spectrophotometer (Shimadzu, Milan, Italy).

### 2.9. Sample Preparation for HPLC Analysis

A solution of 1 mg/mL of ACE dissolved in the mobile phase was prepared for the detection of anthocyanins, while a more concentrated solution (2.5 mg/mL) was prepared for the detection of flavonols, phenolic acids, and other phenolic compounds. The solutions were filtered through a PTFE (0.45 µM) filter before HPLC analysis.

### 2.10. HPLC-ESI-MS, MS^2^ and UV/DAD Analyses

HPLC-ESI-MS and MS^2^ analyses were carried out by using an Agilent Technologies modular 1200 system, equipped with a vacuum degasser, a binary pump, a thermostated autosampler, a thermostated column compartment, and a 6310A ion trap mass analyzer with an ESI ion source (Waldbronn, Germany). The HPLC analysis was carried out on a Zorbax SB-C_18_ column (150 × 4.6 mm I.D., 5 μm, Agilent Technologies) [32]. The mobile phase was composed of (A) H_2_O-HCOOH (9:1, *v*/*v*) and (B) MeOH-H_2_O-HCOOH (5:4:1, *v*/*v*/*v*) and eluted under the following gradient: 0–20 min from 10 to 60% B and 20–25 min from 60 to 80% B. The post-running time was 5 min. The flow rate was 1.0 mL/min, which was split 5:1 before the ESI source. The column temperature was set at 25 °C. The injection volume was 5 μL for anthocyanin analysis and 10 μL for the analysis of all the other phenolics, respectively. 

The ion trap mass analyzer was operated both in the positive and negative ion mode. The experimental parameters were set as follows: the capillary voltage was ±3.5 kV, the nebulizer (N_2_) pressure was 32 psi, the drying gas temperature was 350 °C, the drying gas flow was 10 L/min, and the skimmer voltage was 40 V. Data were acquired using the Agilent 6300 Series Ion Trap LC/MS system software (version 6.2). The mass spectrometer was operated in the full-scan mode in the m/z range 100–1000. MS^2^ spectra were automatically performed with helium as the collision gas in the m/z range 50–1000 with the SmartFrag function.

HPLC-UV/DAD analyses were performed on an Agilent Technologies (Waldbronn, Germany) modular model 1100 system, consisting of a vacuum degasser, a quaternary pump, an autosampler, a thermostated column compartment, and a diode array detector (DAD). Chromatograms were recorded using an Agilent Chemstation for LC and LC-MS systems (Rev. B.01.03). The HPLC column and the applied chromatographic conditions were the same as those reported for the HPLC-ESI-MS system. The UV/DAD acquisitions were carried out in the range 190–650 nm and chromatograms were recorded 520 and 280 nm for anthocyanins and phenolic acids/flavonoids, respectively. The injections were performed in triplicate for each sample.

### 2.11. Cell Cultures

Murine BV2 microglia cells were cultured in RPMI 1640 containing glutamine (2 mM), penicillin (100 U/mL), streptomycin (100 µg/mL) supplemented with 10% FBS. Human neuroblastoma SH-SY5Y cells were cultured in 1:1 mixture of DMEM-F12 Medium, containing glutamine (2 mM), penicillin (100 U/mL), streptomycin (100 µg/mL), non-essential aminoacids 1%, supplemented with 10% FBS.

Both cell lines were incubated in a humidified atmosphere containing 5% CO_2_ at 37 °C, maintained at the confluence of 90% for BV2 and 70% for SH-SY5Y and then subjected to no more than 20 cell passages.

### 2.12. Cell Viability

BV2 and SH-SY5Y cells were seeded in 96-well plates at 5000 and 25,000 cells/well, respectively. ROT was used to induce cytotoxicity effects in both cell lines after preliminary concentration-effect experiments (data not shown). ACE at different concentrations (0.5–50 µg/mL) was then assessed for its cellular effect on viability. To evaluate potential protective effects of ACE against ROT-induced cytotoxicity, cells were pre-treated either with ACE (1–50 µg/mL for BV2 and 0.5–5 µg/mL for SH-SY5Y) or control medium (with 5% FBS) for 24 h, then treated with ACE, ROT, ROT + ACE or control medium. ROT concentrations applied were 0.2 µM for BV2 and 5 µM for SH-SY5Y cells, respectively. Cell viability assays were performed with the MTT test; briefly, formazan crystals produced were solubilized by SDS and the absorbance was read at 570 and 620 nm (as reference wavelength). 

### 2.13. Determination of Reactive Oxygen Species (ROS) in Cells

BV2 and SH-SY5Y cells (10,000 or 50,000 cells/well, respectively) were seeded in a 96-well plate and allowed to adhere overnight. Cells were then pre-treated with either ACE or control medium for 24 h. Afterwards, they were washed once with HBSS and incubated for 45 min at 37 °C with 10 µM H2DCFDA dye. After incubation, dye was removed and cells treated for 8 h with ROT, ACE, ROT + ACE or control medium. ROT concentrations used were 5 µM for BV2 and 15 µM for SHSY-5Y cells, respectively, while ACE was always used at 10 µM for both cell lines. Cell staining and treatment were performed in HBSS and the plates were read on a plate scanner (Fluoroskan FL Microplate Fluorometer, Thermo Fisher Scientific, Waltham, MA, USA) using 485/520 nm excitation/emission wavelengths.

### 2.14. Non-Contact Co-Culture Assay with BV2 and SH-SY5Y Cells

SH-SY5Y cells were seeded in 96-well plates and allowed to adhere for 24 h. BV2 cells were plated in 24 well plates and treated with ROT (0.2 µM), ACE (5–10 µg/mL), ROT (0.2 µM) + ACE (5–10 µg/mL) and control medium for 24 h. Media from each treatment were collected and centrifuged at 780× *g* for 5 min. After centrifugation, BV2 cell supernatants were used to treat SH-SY5Y cells for 24 h. Cellular viability of SH-SY5Y cells was determined using the MTT assay.

### 2.15. Drosophila melanogaster Strains and Maintenance

White-eyed (*w1118)* strain flies were reared on Jazz-Mix Drosophila Food supplemented with Yeast *Kluyveromyces fragilis* at 25 °C with 60% humidity and 12-h light/dark cycle. Approximately 25–30 mating pairs were transferred into food-containing vials and allowed to lay eggs. After nine days, newly enclosed male flies were collected over a period of three days and used in further experiments.

### 2.16. Treatment of Drosophila melanogaster

For behavioural assays, newly enclosed male flies were randomly separated into groups of 50 flies each and transferred to control (medium only) or in treatment vials (media added with ROT 250 µM, ACE 50 µg/mL or ROT + ACE), whose concentration values were considered as final in the media. 

#### 2.16.1. Spontaneous Locomotor Activity

Spontaneous locomotor activity was monitored using the Drosophila Activity Monitoring System (DAMS, Trikinetics, Waltham, MA, USA). To perform this analysis, flies treated for seven days with the above-mentioned substances were housed individually in monitor glass tubes. At one end of the tube, control food and a cap were placed, while cotton was placed at the other for the air passage. Lights were set a 12-h light/dark cycle and least 32 individual flies were tested in each treatment group. The locomotor activity was recorded every minute for 24 h.

#### 2.16.2. Short-Term Shock-Induced Locomotor Activity-Negative Geotaxis (Climbing)

For this assay, 20–30 adult male flies were anesthetized and placed in a vial (length, 12 cm; diameter, 3.0 cm) with the indication of three height ranges top, medium, and bottom. After a brief recovery period, flies were gently tapped to the bottom of the vial. During 5 sec, flies were filmed and the number of them that reached each the three-height section were counted separately; this procedure was repeated five times for experiment. Only top section results, being the most significant ones, are shown in the present work for the sake of clarity.

#### 2.16.3. Longevity Assay

Males of *D. melanogaster*, 1- to 3-day old, were used for this assay. Fifty flies per vial (three replicates per group) were exposed to media containing either the treatment or the control. Flies were changed at least once per week into new vials, containing the same concentrations of the treatment. Flies were monitored and mortality was registered every two days for 60 days and used to plot the longevity curves.

### 2.17. Determination of Drosophila Reactive Oxygen Species (ROS)

Heads of 15 flies were obtained from each treatment group and homogenized in sodium-phosphate buffer (0.1 M; pH 7.4), followed by centrifugation (2500× *g* for 10 min at 4 °C). The supernatant was evaluated for protein content by Bradford method and 20 µg of protein for each sample was used to estimate ROS levels. Briefly, H2DCFDA (5 µM, final concentration) was added to 100 µL of sample in a 96 well plate; after 45 min, fluorescence was read at 490/522 nm.

### 2.18. Maintenance Conditions for Caenorhabditis elegans

Wild type N2 *C. elegans* strains were routinely propagated at 20 °C on nematode growth medium (NGM) plates using *E. coli* strain OP50 as the food source, as previously specified [29]. Synchronization of worm cultures was carried out in order to ensure that all individuals to be used for a given test were in the same larval state, or rather, they were all the same age. This was achieved treating gravid hermaphrodites with a solution of sodium hypochlorite and NaOH, as previously reported [29]. After the synchronization process (syncro), the final residue was re-suspended in 1 mL of M9 and incubated for 3 h at 20 °C in a rotating device to facilitate the development of the eggs to the larval state L1. The plates were sowed with about 200–300 µL of synchro and 200 µL of *E. coli* OP50.

### 2.19. Response to Thermal Stress

This assay was carried out according to the method described by Saul et al. [33]. The procedure requires the use of worms that grew from the larval stage L1 until they reached the 2nd and the 9th day of adulthood. ACE, dissolved in DMSO, was added to the nematode growth medium during its preparation to obtain appropriate concentrations in the plates. On the 1st and 8th day of adulthood, treated (25 µg/mL) and control worms were individually transferred to Petri plates Ø 35 mm previously inoculated 20 µL of *E. coli* OP50. About 20 worms were transferred to each plate with the appropriate treatment, taking care not to break the agar medium. The day after, 2nd or 9th day of adulthood, worms in these little plates were submitted to thermal stress (6 and 8 h at 35 °C). Afterwards, dead and alive nematodes were counted at 6 and 8 h. Worms were considered dead when they did not move or react to stimulus such as slight touches with platinum wire. Assay were performed with approximately 100 nematodes per treatment, and the experiment was done in triplicate. The results were expressed as a percentage of living worms in each assay.

### 2.20. Statistical Analysis

Each experiment was performed at least three times, and all values are represented as mean ± standard error of the mean (SEM). One-way analysis of variance (ANOVA) was used to compare differences among groups, followed by Bonferroni test (Prism 8; GraphPad Prism Software, San Diego, CA, USA). Values of *p* < 0.05 were considered statistically significant. 

The Kaplan–Meier method was used to compare the survival curves of *D. melanogaster* and the survival differences were tested for statistical significance using the log rank test (Mantel Cox).

To analyze survival against thermal stress in the assays with *C. elegans*, contingency tables were prepared, and statistical significance was calculated using the Chi Square Test. In every analysis, significant differences were statistically considered at the level of *p* < 0.05.

## 3. Results and Discussion

### 3.1. Identification and Quantification of Polyphenols, Anthocyanins and Flavonoids

The content in total polyphenols, anthocyanins, and flavonoids of fresh sweet cherry fruit and ACE were assessed by means of colorimetric assays. For what concerns the fresh fruit, total polyphenols (TP) content was found to be 2.5 ± 0.2 mg_GAE_/g (FW), while total anthocyanins (TA) amounted 1.2 ± 0.2 mg_Cy-gluE_/g (FW). Cherry fruits were found to contain a low amount of total flavonoids, it being 0.01 mg_QE_/g (FW). 

A different situation was observed for ACE, as TP amounted to 403.6 ± 41.7 mg_GAE_/g, while TA and TF contents were found to be 167.7 ± 19.2 mg_Cy-gluE_/g and 2.2 ± 0.7 mg_QE_/g, respectively. These results agree with previous papers for what concerns the investigated compounds in sweet cherries [18,34,35]. An average concentration of total polyphenols of 135 mg GAE/100 g FW was reported by Mozetic et al. in five different cultivars of sweet cherries from the Nova Gorica Region (Slovenia) [27], while Matias et al. found a total phenolic content of 456.9 mg GAE/g of dry extract in a Portuguese variety of cherries [35]. 

**Figure 1:**
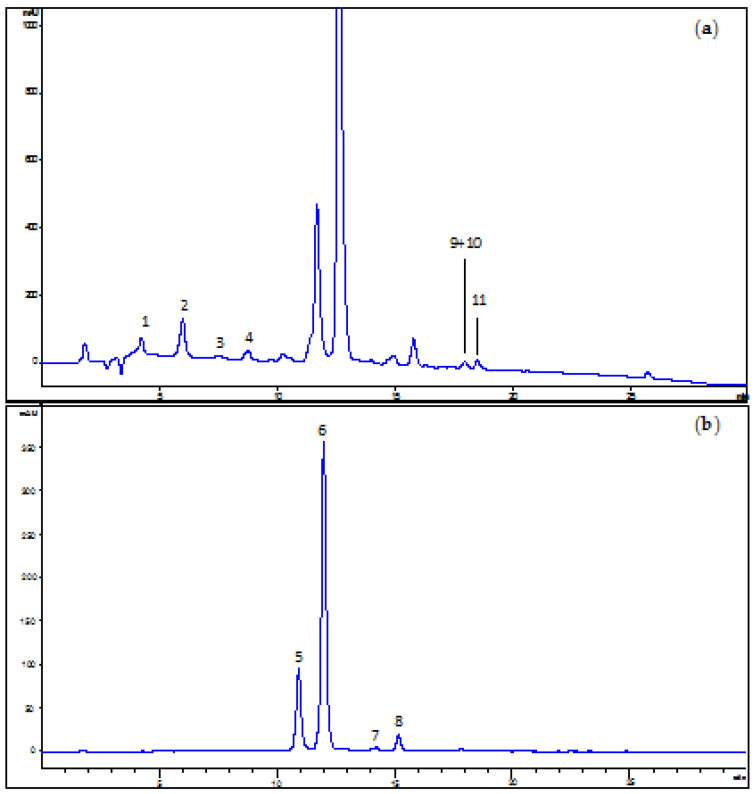
Representative HPLC-UV/DAD chromatogram of the anthocyanin-rich extract (ACE) from sweet cherries dissolved in the mobile phase and recorded at 280 nm (**a**) for the detection of phenolic acids and flavonols, and at 520 nm (**b**) for the detection of anthocyanins. For peak identification, see Table 1.

ACE was further characterized for its individual composition in polyphenolic compounds based on HPLC, UV/Vis, MS, and MS^2^ data compared with standards commercially available and data in the existing literature [22,23,36,37,38,39]. The HPLC-ESI-MS and MS^2^ experiments were carried out both in the positive ion mode for anthocyanins and in the negative ion mode for other flavonoids and phenolic acids. Representative HPLC-UV/DAD chromatograms of an ACE solution in acidified MeOH recorded at 280 and 520 nm are shown in Figure 1, while in Table 1 the identified compounds are listed. 

Three main chemical classes of compounds were determined, namely hydroxycinnamic acids, flavonols, and anthocyanins. Anthocyanins were the predominant type of phenolic compounds in the extracts, mostly represented by cyanidin-3-*O*-rutinoside (**6**). Cyanidin-3-*O*-glucoside (**5**), pelargonidin-3-*O*-rutinoside (**7**), and peonidin-3-*O*-rutinoside (**8**) are other minor anthocyanins found in ACE. Semi-quantitative data of single anthocyanin contents based on the peak areas recorded in the chromatograms led to the following percentages: 20.6 ± 0.5%, 71.9 ± 1.0%, 0.9 ± 0.1% and 3.6 ± 0.1% for (**5**), (**6**), (**7**), and (**8**), respectively. Among hydroxycinnamic acids, 3-coumaroylquinic acid (**2**) represented the most abundant one, followed by 4-coumaroylquinic acid (**4**) and neochlorogenic acid (**1**). Other minor phenolic acids were also detected, including feruloylquinic acid (**3**) and dicaffeoylquinic acid (**9**). As to flavonols, the 3-*O*-glucoside (**10**) and 3-*O*-rutinoside (**11**) of quercetin were identified.

### 3.2. Effect of ACE on Rotenone-Induced Cytotoxicity in Microglia BV2 Cells

**Figure 2:**
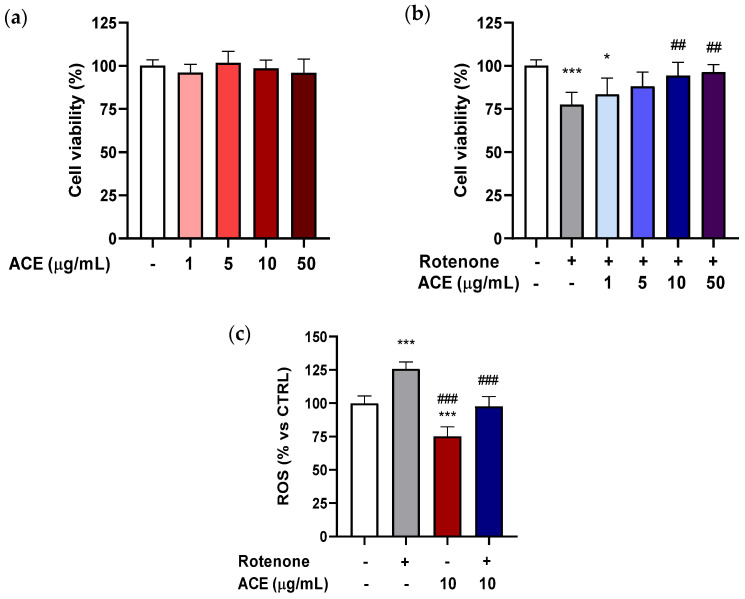
Effects of ACE (1–5–10–50 μg/mL) on cellular viability and reactive oxygen species (ROS) levels in BV2 cells. (**a**) Effects on BV2 cellular viability by ACE alone; (**b**) by ACE after ROT-induced BV2 cell toxicity; and (**c**) on ROS levels after BV2 cell exposure to ROT. All data are expressed as mean ± standard error (*n* = 3). Significance was assessed by one-way analysis of variance (ANOVA), followed by Bonferroni multiple comparison test, as compared to control (white bar) at *p* < 0.05 (*) and *p* < 0.001 (***), and as compared to toxic agent at *p* < 0.01 (^##^) and *p* < 0.001 (^###^).

In this study, BV2 cells were grown for 24 h in 96-well plates, and subsequently treated with ACE at different concentrations (1–50 µg/mL) for the same time period. As shown in Figure 2a, the extract, in the range of concentrations examined, was not able to significantly influence the viability of the BV2 cells.

To explore the possibility that the extract was able to exert a neuroprotective action on microglia, the ability of the extracts to significantly modify the cytotoxicity of BV2 cells, using MTT test was tested. BV2 cells were pre-treated for 24 h with ACE at the concentrations studied, and finally, for another 24 h with ROT + ACE. ROT at 0.2 µM (concentration capable of significantly decreasing the vitality of BV2 cells by about 20%) and ACE 1–50 µg/mL (range of concentrations that had proved ineffective per se in increasing viability of the microglia cells) were co-applied. As shown in Figure 2b, when co-applied to ROT, ACE was able to decrease toxicity, starting from a concentration of 5 µg/mL, and to reinstate cell viability to values similar to those of the control at concentrations of 10 and 50 µg/mL. It should be highlighted that microglial cells, such as BV-2, are extremely sensitive to the action of ROT, which, applied at a concentration of 0.2 µM for 24 h, proved sufficient to significantly decrease the vitality of these cells. 

These results are in agreement with those carried out on *Mucuna pruriens* seed extracts, which reduced hydrogen peroxide-induced cytotoxicity in BV2 cells [40].

### 3.3. Effect of ACE on Rotenone-Induced Augmentation of Reactive Oxygen Species (ROS) Production in Microglia BV2 Cells

The protective effect of ACE (10 μg/mL) against the production of ROS by ROT (5 µg/mL) in BV2 cells was evaluated after 8 h exposure. As shown in Figure 2c, the production of ROS in ROT-treated BV2 cells was elevated by 26% as compared with control cells. ACE reduced the ROT-induced production of ROS by 25% and the combination ROT + ACE was able to revert the ROT-induced increase of ROS, restoring the values to those of controls. 

It has been previously shown that anthocyanin-rich extracts from berries were able to reduce inflammatory and oxidative stresses in BV-2 microglia [41], which is in line with similar effects reported for anthocyanins from other berries [42].

### 3.4. Effect of ACE on Rotenone-Induced Decrease in SH-SY5Y Cell Viability

**Figure 3:**
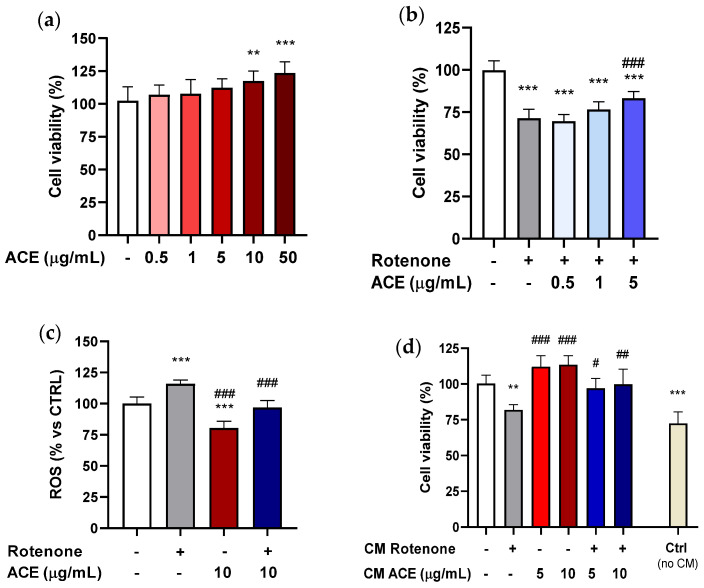
Effects of ACE (1–5–10–50 μg/mL) on cellular viability and reactive oxygen species (ROS) levels in SH-SY5Y cells. (**a**) Effects on SH-SY5Y cellular viability by ACE alone; (**b**) by ACE after ROT-induced SHSY-5Y cell toxicity; and (**c**) on ROS levels after SHSY-5Y cell exposure to ROT; (**d**) effects on SH-SY5Y cell viability after non-contact co-culture with conditioned media (CM) from BV2 treated with ROT and/or ACE. All data are expressed as mean ± standard error (*n* = 3). Significance was assessed by one-way analysis of variance (ANOVA), followed by Bonferroni multiple comparison test, as compared to control (white bar) at *p* < 0.01 (**) and *p* < 0.001 (***), and as compared to toxic agent at *p* < 0.05 (^#^), *p* < 0.01 (^##^) and *p* < 0.001 (^###^).

SH-SY5Y cells were grown for 24 h in 96-well plates and subsequently treated with ACE at different concentrations (0.5–50 µg/mL) for the same time period. As it can be seen from Figure 3a, the extract, administered alone, was able to increase cell viability only at the highest concentrations (10 and 50 µg/mL).

To verify whether the concentrations of extract used may exert a neuroprotective action on SH-SY5Y cells, they were treated by co-applying the minimal effective dose of ROT (5 µM; −20% vs. CTRL), deriving from previous experiments where increasing concentrations of this compound were used (data not shown), and ACE, at the concentrations that had been demonstrated to be ineffective in producing a significant increase in cell viability (0.5–5 µg/mL). SH-SY5Y cells were also pre-treated for 24 h with the three predetermined concentrations of extract, and finally for additional 24 h with the association of ROT and ACE.

For SH-SY5Y cells, ACE at the highest concentration used (5 µg/mL) was capable of significantly increasing viability compared to cells treated with ROT alone, but it did not bring the values back to the control levels, thus giving an intermediate effect, as can be seen in Figure 3b. If compared to BV2 microglia, SH-SY5Y cells appear to be more resistant to the action of ROT: the concentration of 5 µM applied for 24 h reduces cell viability by about 25%. 

Neuron-like SH-SY5Y cells have been previously treated with various cherry extracts, with differential effects in counteracting oxidative stress [34].

### 3.5. Effetc of ACE on Rotenone-Induced ROS Enhancement in SH-SY5Y Cells

To better characterize the protection mechanisms, the ability of ACE to counteract ROT-induced intracellular ROS production in SH-SY5Y cells was investigated, using the DCFH-DA assay, after 8 h exposure. As shown reported in Figure 3c, ACE (10 µg/mL) per se led to a considerable reduction in intracellular ROS levels (−25%), while ROT (15 µg/mL) significantly increased them (+20%). The combination of ACE and ROT was able to bring back the ROS production to that of controls. As mentioned above, ROT acts as a selective inhibitor of complex I of the mitochondrial electronic transport chain, increasing the production of oxygen free radicals and thus inducing strong oxidative stress within neurons [43].

### 3.6. Effect of ACE on SH-SY5Y Cells in a Non-Contact Co-Culture Model (Using Conditioned Media from BV2 Cells)

To confirm that BV2 cells treated with ROT can induce factors with neuroprotective/neurotrophic action on SH-SY5Y cells, they were treated with conditioned media (CM) derived from BV2 cells previously exposed to ROT (0.2 µM), ACE (5 and 10 µg/mL) and with the combination of the two. In this regard, BV2 cells were grown in 24-well plates for 24 h, and then treated for further 24 h in the above-mentioned way. Afterwards, the conditioned culture media were removed from the BV2 cells and used for the treatment of SH-SY5Y cells for additional 24 h.

Figure 3d illustrates that CM resulting from the treatment with ROT showed a significant level of toxicity compared to CM control (with conditioned medium deriving from control BV2 cells) when administered to SH-SY5Y cells, while the CM from BV2 cells treated with the two concentrations of ACE did not produce significant alterations. On the contrary, the CM from BV2 treated with ROT + ACE was able to reduce toxicity at either concentration, suggesting a neuroprotective role of the extract itself. Besides, control SH-SY5Y cell viability, without adding CM, revealed to be significantly lower with respect to that of CM controls.

It was observed that treatment of SH–SY5Y cells with some of the conditioned culture media improved their viability, as it was the case for CM derived from control BV2 cells (without any treatment). These data seem to support the hypothesis that BV2 cells not previously exposed to toxic insult are able to produce factors that could in some way improve the vitality of neuroblastoma cells.

These results are in agreement with other work that evaluated the effect of CM from BV-2 cells on SH-SY5Y cells for other classes of protective compounds [43,44]. 

### 3.7. Effect of ACE on Locomotion Impairment and Lethality in Rotenone-Induced Neurotoxicity in Drosophila melanogaster

On the basis of the encouraging results obtained in the in vitro model of PD in vitro, further assays were performed to assess the effects of ACE using an in vivo model based on *D. melanogaster*. Two methods for measuring motor activity in *Drosophila* were compared: locomotion induced by short-term mechanical insult and long-term spontaneous locomotion, in order to better understand the effects of motor deficiencies mediated by ROT.

#### 3.7.1. Long-Term Spontaneous Locomotor Activity

Male flies were placed in individual tubes and the recording of movements was detected by the DAMS monitoring system. This appliance is equipped with an infrared sensor, which counts the number of times a fly interrupts the infrared beam, located in the middle of the tube. These counts are considered as a measure of undisturbed locomotion and activity. Thus, the effect of ROT on long-term locomotion (24 h) was evaluated.

**Figure 4:**
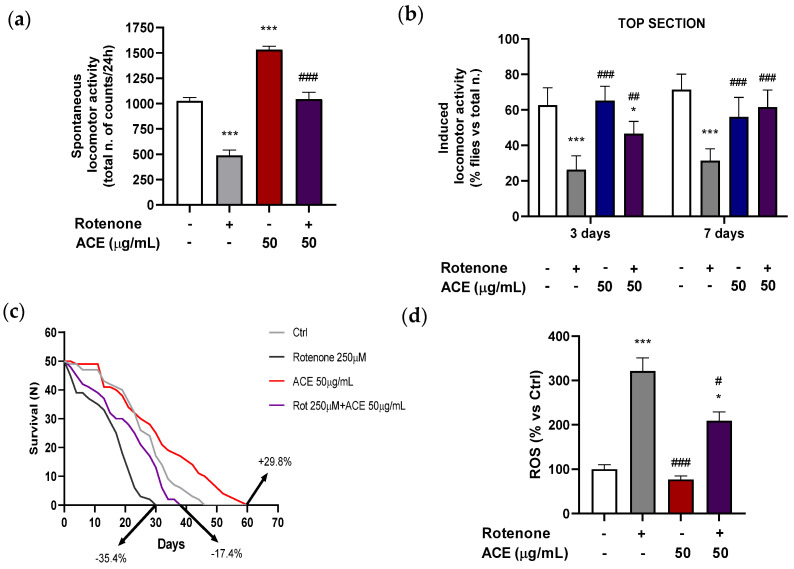
Effects of ACE (50 µg/mL) and/or ROT (250 µM): (**a**) in adult male of *Drosophila melanogaster* on spontaneous locomotion (24 h) registered with DAMS; (**b**) on negative geotaxis (3rd or 7th treatment day), with bars indicating the percentage of flies that climb to the top of vial within 5 sec (*n* = 5); (**c**) on lifespan (*n* = 50; fly survival rate) after 60 days of treatment (the percentage in the figure represents the comparison with control group survival rate); (**d**) on ROS levels measured in flies head homogenate. Survival curves were statistically analyzed by log rank test (Mantel Cox). Significance was assessed by one-way analysis of variance (ANOVA), followed by Bonferroni multiple comparison test, as compared to control (white bar) at *p* < 0.05 (*) and *p* < 0.001 (***), and as compared to toxic treatment (with three replicates of *n* = 25) at *p* < 0.05 (^#^), *p* < 0.01 (^##^) and *p* < 0.001 (^###^).

Flies, after seven days of exposure to ROT, showed a tendency towards a reduction in locomotor activity levels that reached significance for the utilized dose of 250 μM, indicating a deficit, induced by the neurotoxin, in spontaneous locomotion. On the contrary, an increase in locomotor activity in the 24 h was evident in the DAMS system in flies treated with 50 μg/mL ACE, with a relevant rise of the total number of movements. Figure 4a shows the spontaneous locomotor activity of *Drosophila* exposed to ROT (250 μM), to ACE (50 μg/mL) and to their associations. When flies were treated simultaneously with ROT and ACE, the total number of movements was significantly improved if compared to that of flies exposed to ROT alone, bringing back the total number of movements in 24 h to values comparable to those of the controls.

Indeed, by inducing the symptoms of PD in *D. melanogaster*, it is possible to better understand the disease and to counteract its symptoms. In this sense, *Drosophila* represents a good model to study this effect because it is genetically modifiable, easy to maintain, and has a rapid cycle [45].

#### 3.7.2. Short-Term Shock-Induced Locomotor Activity (Climbing)

*Drosophila* showed a clear negative geotactic response, with approximately 50–60% of total individuals present, after 5 sec, in the upper (top) section of the “climbing” apparatus (elective parameter to indicate alterations in the locomotor activity). A dose of 250 μM ROT was chosen, as it showed a significant decrease in the percentage of individuals in the upper section. This “defect” in the negative geotactic response due to the inability to climb rapidly in the apparatus is indicative of a deficit in locomotion, induced by the mechanical insult.

Moreover, a dose of 50 µg/mL ACE was selected for the present experiments, as it revealed to be the lower effective one able to increase the percentage of flies in the top section at either day tested.

When the combined effects of ACE (50 µg/mL) with ROT (250 µM) in the nutrient medium on *Drosophila* in the upper section were examined after a three-day treatment (Figure 4b), it was found that ACE alone provided an improvement trend in induced locomotor activity, although not significant, while ROT determines a marked deficit in motor performance, with respect to controls. ACE combined with ROT improved the performance of *Drosophila* in negative geotaxis, being effective in bringing the percentages to intermediate levels although significantly different if compared with ROT-treated ones.

After seven-day administration, ACE (50 µg/mL) *per se* was unable to determine significant changes in % values in the top section while, in combination with ROT, it was capable of reverting the decrease in % flies in top section, induced by ROT alone, restoring the values to those of controls (Figure 4b). 

This reversal of the trend of ACE at seven days with respect to induced locomotion with the progress of time, compared to three-day treatment, is probably due to an excessive concentration of the extract in the nutrient medium, which leads to effects that we can consider somehow counterproductive in relation to the locomotion “performance”.

It is important to note that, by administering ROT and ACE together, the association improved the deficit caused by ROT in negative geotaxis. Several studies have shown that ROT causes short-term shock-induced locomotion defects in *Drosophila*. When flies are kept on food supplemented with ROT, they exhibit a slower negative geotactic response after mechanical insult [46,47].

In agreement with our results, several studies have shown that exposure to ROT in *D. melanogaster* induces the fundamental characteristic of PD, i.e., the deficit of locomotor behavior in the negative geotaxis test. For the first time, Siima et al. demonstrated that flavonoids derived from *Erythrina schliebenii* mitigated the “climbing” disability caused by ROT [48]. A study by Pandareesh et al. with curcumin has shown that the integration of the latter in the diet of *Drosophila* improved the damage recorded in the negative geotaxis test and in survival experiments [49]. Finally, extracts of the Indian aquatic plant Bacopa, when co-administered with the neurotoxin, led to an improvement in mortality and negative geotaxis in the ROT-induced model of PD in *Drosophila* [50].

#### 3.7.3. Longevity Curves

Exposure of adult flies (*n* = 50) to increasing ROT concentrations (62.5–500 μM) resulted in a dose-dependent reduction in lifespan, when compared to the mean number of days (48) in the group of vehicle-treated control (DMSO) (data not shown). Longevity curves showed a concentration-dependent increase in mortality values for ROT, while different concentrations of ACE (1–100 μg/mL in the diet) prolonged the lifespan of *D. melanogaster* in a concentration-dependent manner.

The co-administration in the diet of 250 μM ROT and 50 μg/mL ACE was effective in significantly reducing the incidence of mortality induced by ROT in flies, settling on intermediate values (−17.4% compared to the control) between the increase in the mortality rate from ROT alone (−35.4%) and the increase in survival produced by ACE alone (+29.8%) (Figure 4c). 

The treatment with ROT significantly reduced the median and maximum survival by 1.5-fold (18 days) and 1.7-fold (24 days), respectively, compared with control flies (28 and 40 days). ACE significantly increased (*p* < 0.05) the median and maximum survival by 1.1-fold (31 days) and 1.3-fold (51 days), respectively, compared with controls. ACE counteracted the effect of ROT when given in combination, significantly increasing the mean and maximum survival in *Drosophila* by 1.3-fold (24 days) and 1.4-fold (34 days), respectively, compared with flies treated with ROT alone (*p* < 0.05).

Several studies have shown that the consumption of diets enriched with substances endowed with antioxidant properties contribute to the prolongation of the lifespan in organisms, including *Drosophila* [46,48,49]. Under our experimental conditions, the exposure of adult flies to ROT caused a reduction in the lifespan when compared with the control group and, in parallel, in the number of days in which 50% of mortality occurred. On the contrary, ACE prolonged the lifespan of *Drosophila* and it increased the average number of days in which 50% of mortality was found.

The beneficial effects of the Moretta extracts obtained on the lifespan in *Drosophila* might be explained by the antioxidant action of ACE, which contains a high component of anthocyanins with distinct antioxidant properties, possibly resulting in the suppression of free radicals and/or in an increased regulation of antioxidant defenses.

### 3.8. Effect of ACE on Oxidative Stress in the Rotenone Fly Model

Flies exposed to ROT (250 µM) treatment exhibited markedly increased ROS (head region: 321.8 ± 30.6%) levels compared to control group (100 ± 11.2%) (Figure 4d). The combination with ACE effectively scavenged the free radicals induced by ROT treatment in the fly head regions, significantly setting the ROS content to intermediate values between those of the controls and ROT (209.3 ± 20.8%, *p* < 0.05 vs. CTRL and vs. ROT) (Figure 4d).

Regarding ROS accumulation, flies treated with the ACE extract showed in head regions a non-significant slight decrease in ROS levels at 50 μg/mL (76.8 ± 7.96%, *p* > 0.05), in relation to controls.

Rao et al. performed similar experiments administering ROT to *Drosophila* males in order to induce Parkinson-like effects and, subsequently, they performed treatments with saffron, rich in polyphenols and antioxidant compounds [51]. The obtained results suggested that the saffron treatment was able to reduce the production of oxygen free radicals playing a protective role against oxidative stress. 

Recently, ROS have emerged as key players in normal cellular signaling by oxidizing redox-sensitive proteins. Therefore, while high levels of ROS may be harmful and induce oxidative stress, low levels of ROS may actually be beneficial as mediators of redox signaling [52,53].

### 3.9. Effect of ACE on Survival in Thermally-Induced Oxidative Stress Model in Caenorhabditis elegans

**Figure 5:**
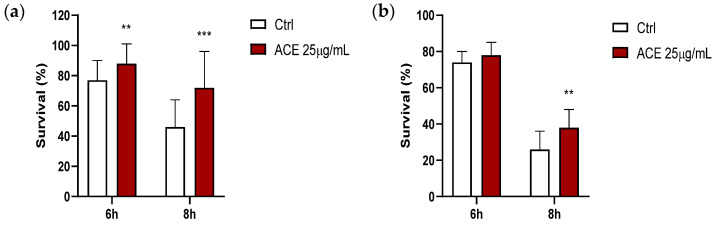
Percentages of worm survival at the 2nd (**a**) and 9th (**b**) day of adulthood after application of thermal stress (35 °C, 6 h or 8 h) in the *Caenorhabditis elegans* wild-type N2 strain grown either in the absence (controls; Ctrl) or presence of ACE. The results are shown as the mean values ± SEM at *p* < 0.01 (**) and *p* < 0.001 (***).

From dose-response curves previously performed, the concentration of ACE that provided a significant increase in the survival of nematodes submitted to thermal stress was 25 μg/mL (corresponding to 5 μg GAE/mL). As shown in Figure 5a, the survival of worms that were cultivated in the presence of ACE significantly increased at the 2nd day of adulthood in relation to controls (DMSO), either after 6 or 8 h of thermally induced oxidative stress, and similarly happened in old adults (9th day of adulthood), although the result was only significant after 8 h of thermal stress, at which there was an increase of 25% in the number of surviving nematodes.

Previous results obtained with a *Vaccinium myrtillus* L. extract on *C. elegans* following thermal stress showed that a low extract concentration (such as 5 μg/mL corresponding to 2.5 μg equivalent of delphinidin/mL) was able to increase survival in both young and old nematodes, while 10 μg/mL of the extract (corresponding to 5 μg equivalent of delphinidin/mL) showed adverse effects [29]. In that case, the difference in survival between control nematodes and those treated with the extract varied between 10 and 15%, while in the present study with the cherry extract, the survival rates ranged between 11 and 25%, thus showing a higher protective capacity for the nematodes after exposure to thermal stress. In agreement with our findings, Jayarathne et al. have found that a tart cherry (*Prunus cerasus* L.) extract conferred health span benefits in *C. elegans*, reducing oxidative stress and enhancing mitochondrial function, mainly via the DAF-16 pathway [54]. Similar results about extension of lifespan after exposure to TCE have described by Van de Klashorst et al., although in this case, the authors postulated that TCE may be operating as a calorie restriction mimetic via metabolic pathways [55].

## 4. Conclusions

In this study, the antioxidant and neuroprotective properties of a cherry extract rich in anthocyanins (ACE), derived from the Moretta di Vignola variety (*Prunus avium* L.), were assessed. This extract demonstrated to be effective at a cellular level in reducing cytotoxicity, intracellular ROS production in two assayed cell lines (i.e., SH-SY5Y and BV2), after exposure to the neurotoxin ROT. Moreover, ACE protected SH-SY5Y cells in a non-contact co-culture with BV2 cells, indicating a cross-talk between the two types of cells in an in vitro model of PD. In addition, for the first time, this cherry extract not only counteracted the behavioral effects of the neurotoxin ROT, but it also mitigated ROT-induced mortality by extending the lifespan in the PD model of *D. melanogaster*. Furthermore, ACE also ameliorated biochemical (ROS) parameters in *Drosophila* after the neurotoxin-induced insult. By combining the data from both the in vitro and in vivo experiments, ACE showed neuroprotective effects in our PD models. In addition, ACE improved the resistance of the nematode *C. elegans* against thermally induced oxidative stress, especially in young animals, suggesting it to be suitable for protection against oxidative stress.

It must be taken into account that the composition and content of polyphenols in fruit extracts from plants, including *P. avium*, are strongly dependent on genetic variability and they are influenced by climatic, agronomic, and post-harvest conditions. By taking into account all these parameters, a special attention should be addressed to the qualitative and quantitative standardization of the fruit extracts in order to provide a standardized composition of bioactive compounds.

Based on the results obtained in this work, the consumption of a standardized extract of this local cherry variety in a nutraceutical product may offer therapeutic benefits to humans in order to either prevent or delay acute and chronic diseases, such as the neurodegenerative ones and in particular PD, at the base of which there are oxidative-inflammatory processes.

## Figures and Tables

**Table 1:** Compounds identified in the anthocyanin-rich cherry extract (ACE).

Peak N.	*t*_R_ (min)	Compound	λ_max_	[M−H]^−^ (*m*/*z*)	[M]^+^ (*m*/*z*)	MS^2^
**1**	4.2	Neochlorogenic acid	291, 323	353	-	191 (100), 179 (30), 135 (8)
**2**	6.0	3-Coumaroylquinic acid	320	337	-	191 (8), 163 (100), 119 (7)
**3**	7.4	Feruloylquinic acid	310sh, 328	367	-	193 (100), 134 (9)
**4**	8.8	4-Coumaroylquinic acid	320	337	-	173 (100), 163 (8)
**5**	11.6	Cyanidin-3-*O*-glucoside	280, 509	-	449	287 (100)
**6**	12.6	Cyanidin-3-*O*-rutinoside	280, 518	-	595	449 (31), 287 (100)
**7**	14.8	Pelargonidin-3-*O*-rutinoside	-	-	579	433 (40), 271 (100)
**8**	15.7	Peonidin-3-*O*-rutinoside	277, 509	-	609	463 (38), 301 (100)
**9**	17.9	Dicaffeoylquinic acid	291, 323	515	-	353 (100), 179 (5)
**10**	18.0	Quercetin-3-*O*-glucoside	268, 366	463	-	301 (100), 211 (7)
**11**	18.5	Quercetin-3-*O*-rutinoside	255, 363	609	-	343 (13), 301 (100), 271 (16), 255 (17)

## Data Availability

Data is contained within the article.
